# Incorporating progesterone receptor expression into the PREDICT breast prognostic model

**DOI:** 10.1016/j.ejca.2022.06.011

**Published:** 2022-09

**Authors:** Isabelle Grootes, Renske Keeman, Fiona M. Blows, Roger L. Milne, Graham G. Giles, Anthony J. Swerdlow, Peter A. Fasching, Mustapha Abubakar, Irene L. Andrulis, Hoda Anton-Culver, Matthias W. Beckmann, Carl Blomqvist, Stig E. Bojesen, Manjeet K. Bolla, Bernardo Bonanni, Ignacio Briceno, Barbara Burwinkel, Nicola J. Camp, Jose E. Castelao, Ji-Yeob Choi, Christine L. Clarke, Fergus J. Couch, Angela Cox, Simon S. Cross, Kamila Czene, Peter Devilee, Thilo Dörk, Alison M. Dunning, Miriam Dwek, Douglas F. Easton, Diana M. Eccles, Mikael Eriksson, Kristina Ernst, D. Gareth Evans, Jonine D. Figueroa, Visnja Fink, Giuseppe Floris, Stephen Fox, Marike Gabrielson, Manuela Gago-Dominguez, José A. García-Sáenz, Anna González-Neira, Lothar Haeberle, Christopher A. Haiman, Per Hall, Ute Hamann, Elaine F. Harkness, Mikael Hartman, Alexander Hein, Maartje J. Hooning, Ming-Feng Hou, Sacha J. Howell, ABCTB Investigators, kConFab Investigators, Hidemi Ito, Anna Jakubowska, Wolfgang Janni, Esther M. John, Audrey Jung, Daehee Kang, Vessela N. Kristensen, Ava Kwong, Diether Lambrechts, Jingmei Li, Jan Lubiński, Mehdi Manoochehri, Sara Margolin, Keitaro Matsuo, Nur Aishah Mohd Taib, Anna Marie Mulligan, Heli Nevanlinna, William G. Newman, Kenneth Offit, Ana Osorio, Sue K. Park, Tjoung-Won Park-Simon, Alpa V. Patel, Nadege Presneau, Katri Pylkäs, Brigitte Rack, Paolo Radice, Gad Rennert, Atocha Romero, Emmanouil Saloustros, Elinor J. Sawyer, Andreas Schneeweiss, Fabienne Schochter, Minouk J. Schoemaker, Chen-Yang Shen, Rana Shibli, Peter Sinn, William J. Tapper, Essa Tawfiq, Soo Hwang Teo, Lauren R. Teras, Diana Torres, Celine M. Vachon, Carolien H.M. van Deurzen, Camilla Wendt, Justin A. Williams, Robert Winqvist, Mark Elwood, Marjanka K. Schmidt, Montserrat García-Closas, Paul D.P. Pharoah

**Affiliations:** a University of Cambridge, Centre for Cancer Genetic Epidemiology, Department of Oncology, Cambridge, CB1 8RN, UK; b The Netherlands Cancer Institute – Antoni van Leeuwenhoek Hospital, Division of Molecular Pathology, Amsterdam, 1066 CX, the Netherlands; c Cancer Council Victoria, Cancer Epidemiology Division, Melbourne, Victoria, 3004, Australia; d The University of Melbourne, Centre for Epidemiology and Biostatistics, Melbourne School of Population and Global Health, Melbourne, Victoria, 3010, Australia; e Monash University, Precision Medicine, School of Clinical Sciences at Monash Health, Clayton, Victoria, 3168, Australia; f The Institute of Cancer Research, Division of Genetics and Epidemiology, London, SM2 5NG, UK; g The Institute of Cancer Research, Division of Breast Cancer Research, London, SW7 3RP, UK; h University of California at Los Angeles, David Geffen School of Medicine, Department of Medicine Division of Hematology and Oncology, Los Angeles, CA, 90095, USA; i Comprehensive Cancer Center Erlangen-EMN, University Hospital Erlangen, Friedrich-Alexander University Erlangen-Nuremberg (FAU), Department of Gynecology and Obstetrics, Erlangen, 91054, Germany; j National Cancer Institute, National Institutes of Health, Department of Health and Human Services, Division of Cancer Epidemiology and Genetics, Bethesda, MD, 20850, USA; k Lunenfeld-Tanenbaum Research Institute of Mount Sinai Hospital, Fred A. Litwin Center for Cancer Genetics, Toronto, ON, M5G 1X5, Canada; l University of Toronto, Department of Molecular Genetics, Toronto, ON, M5S 1A8, Canada; m University of California Irvine, Department of Medicine, Genetic Epidemiology Research Institute, Irvine, CA, 92617, USA; n University of Helsinki, Department of Oncology, Helsinki University Hospital, Helsinki, 00290, Finland; o Örebro University Hospital, Department of Oncology, Örebro, 70185, Sweden; p Copenhagen University Hospital, Copenhagen General Population Study, Herlev and Gentofte Hospital, Herlev, 2730, Denmark; q Copenhagen University Hospital, Department of Clinical Biochemistry, Herlev and Gentofte Hospital, Herlev, 2730, Denmark; r University of Copenhagen, Faculty of Health and Medical Sciences, Copenhagen, 2200, Denmark; s University of Cambridge, Centre for Cancer Genetic Epidemiology, Department of Public Health and Primary Care, Cambridge, CB1 8RN, UK; t IEO, European Institute of Oncology IRCCS, Division of Cancer Prevention and Genetics, Milan, 20141, Italy; u Universidad de La Sabana, Medical Faculty, Bogota, 140013, Colombia; v German Cancer Research Center (DKFZ), Molecular Epidemiology Group, C080, Heidelberg, 69120, Germany; w University of Heidelberg, Molecular Biology of Breast Cancer, University Womens Clinic Heidelberg, Heidelberg, 69120, Germany; x University of Utah, Department of Internal Medicine and Huntsman Cancer Institute, Salt Lake City, UT, 84112, USA; y Instituto de Investigación Sanitaria Galicia Sur (IISGS), Xerencia de Xestion Integrada de Vigo-SERGAS, Oncology and Genetics Unit, Vigo, 36312, Spain; z Seoul National University Graduate School, Department of Biomedical Sciences, Seoul, 03080, South Korea; aa Seoul National University, Cancer Research Institute, Seoul, 03080, South Korea; ab Seoul National University Medical Research Center, Institute of Health Policy and Management, Seoul, 03080, South Korea; ac University of Sydney, Westmead Institute for Medical Research, Sydney, New South Wales, 2145, Australia; ad Mayo Clinic, Department of Laboratory Medicine and Pathology, Rochester, MN, 55905, USA; ae University of Sheffield, Sheffield Institute for Nucleic Acids (SInFoNiA), Department of Oncology and Metabolism, Sheffield, S10 2TN, UK; af University of Sheffield, Academic Unit of Pathology, Department of Neuroscience, Sheffield, S10 2TN, UK; ag Karolinska Institutet, Department of Medical Epidemiology and Biostatistics, Stockholm, 171 65, Sweden; ah Leiden University Medical Center, Department of Pathology, Leiden, 2333 ZA, the Netherlands; ai Leiden University Medical Center, Department of Human Genetics, Leiden, 2333 ZA, the Netherlands; aj Hannover Medical School, Gynaecology Research Unit, Hannover, 30625, Germany; ak University of Westminster, School of Life Sciences, London, W1B 2HW, UK; al University of Southampton, Faculty of Medicine, Southampton, SO17 1BJ, UK; am University Hospital Ulm, Department of Gynaecology and Obstetrics, Ulm, 89075, Germany; an University of Manchester, Manchester Academic Health Science Centre, Division of Evolution and Genomic Sciences, School of Biological Sciences, Faculty of Biology, Medicine and Health, Manchester, M13 9WL, UK; ao St Mary's Hospital, Manchester University NHS Foundation Trust, Manchester Academic Health Science Centre, North West Genomics Laboratory Hub, Manchester Centre for Genomic Medicine, Manchester, M13 9WL, UK; ap The University of Edinburgh, Usher Institute of Population Health Sciences and Informatics, Edinburgh, EH16 4UX, UK; aq The University of Edinburgh, Cancer Research UK Edinburgh Centre, Edinburgh, EH4 2XR, UK; ar Leuven Cancer Institute, University Hospitals Leuven, Leuven Multidisciplinary Breast Center, Department of Oncology, Leuven, 3000, Belgium; as Peter MacCallum Cancer Center, Melbourne, Victoria, Australia, 3000; at Instituto de Investigación Sanitaria de Santiago de Compostela (IDIS), Complejo Hospitalario Universitario de Santiago, SERGAS, Fundación Pública Galega de Medicina Xenómica, Santiago de Compostela, 15706, Spain; au University of California San Diego, Moores Cancer Center, La Jolla, CA, 92037, USA; av Instituto de Investigación Sanitaria San Carlos (IdISSC), Centro Investigación Biomédica en Red de Cáncer (CIBERONC), Medical Oncology Department, Hospital Clínico San Carlos, Madrid, 28040, Spain; aw Spanish National Cancer Research Centre (CNIO), Human Cancer Genetics Programme, Madrid, 28029, Spain; ax University of Southern California, Department of Preventive Medicine, Keck School of Medicine, Los Angeles, CA, 90033, USA; ay Södersjukhuset, Department of Oncology, Stockholm, 118 83, Sweden; az German Cancer Research Center (DKFZ), Molecular Genetics of Breast Cancer, Heidelberg, 69120, Germany; ba University of Manchester, Manchester Academic Health Science Centre, Division of Informatics, Imaging and Data Sciences, Faculty of Biology, Medicine and Health, Manchester, M13 9PT, UK; bb Wythenshawe Hospital, Manchester University NHS Foundation Trust, Nightingale & Genesis Prevention Centre, Manchester, M23 9LT, UK; bc Manchester University NHS Foundation Trust, Manchester Academic Health Science Centre, NIHR Manchester Biomedical Research Unit, Manchester, M13 9WL, UK; bd National University of Singapore and National University Health System, Saw Swee Hock School of Public Health, Singapore, 119077, Singapore; be National University Health System, Department of Surgery, Singapore, 119228, Singapore; bf Erasmus MC Cancer Institute, Department of Medical Oncology, Rotterdam, 3015 GD, the Netherlands; bg Kaohsiung Municipal Hsiao-Kang Hospital, Department of Surgery, Kaohsiung, 812, Taiwan; bh University of Manchester, Division of Cancer Sciences, Manchester, M13 9PL, UK; bi University of Sydney, Australian Breast Cancer Tissue Bank, Westmead Institute for Medical Research, Sydney, New South Wales, 2145, Australia; bj Peter MacCallum Cancer Center, Research Department, Melbourne, Victoria, Australia, 3000; bk The University of Melbourne, Sir Peter MacCallum Department of Oncology, Melbourne, Victoria, 3000, Australia; bl Aichi Cancer Center Research Institute, Division of Cancer Information and Control, Nagoya, 464-8681, Japan; bm Nagoya University Graduate School of Medicine, Division of Cancer Epidemiology, Nagoya, 466-8550, Japan; bn Pomeranian Medical University, Department of Genetics and Pathology, Szczecin, 71-252, Poland; bo Pomeranian Medical University, Independent Laboratory of Molecular Biology and Genetic Diagnostics, Szczecin, 71-252, Poland; bp Stanford University School of Medicine, Department of Epidemiology & Population Health, Stanford, CA, 94305, USA; bq Stanford Cancer Institute, Stanford University School of Medicine, Department of Medicine, Division of Oncology, Stanford, CA, 94304, USA; br German Cancer Research Center (DKFZ), Division of Cancer Epidemiology, Heidelberg, 69120, Germany; bs Seoul National University College of Medicine, Department of Preventive Medicine, Seoul, 03080, South Korea; bt Oslo University Hospital and University of Oslo, Department of Medical Genetics, Oslo, 0379, Norway; bu University of Oslo, Institute of Clinical Medicine, Faculty of Medicine, Oslo, 0450, Norway; bv Hong Kong Hereditary Breast Cancer Family Registry, Hong Kong; bw The University of Hong Kong, Department of Surgery, Hong Kong; bx Hong Kong Sanatorium and Hospital, Department of Surgery and Cancer Genetics Center, Hong Kong; by VIB Center for Cancer Biology, VIB, Leuven, 3001, Belgium; bz University of Leuven, Laboratory for Translational Genetics, Department of Human Genetics, Leuven, 3000, Belgium; ca Genome Institute of Singapore, Human Genetics Division, Singapore, 138672, Singapore; cb Karolinska Institutet, Department of Clinical Science and Education, Södersjukhuset, Stockholm, 118 83, Sweden; cc Aichi Cancer Center Research Institute, Division of Cancer Epidemiology and Prevention, Nagoya, 464-8681, Japan; cd University of Malaya, Department of Surgery, Faculty of Medicine, Kuala Lumpur, 50603, Malaysia; ce University of Toronto, Department of Laboratory Medicine and Pathobiology, Toronto, ON, M5S 1A8, Canada; cf University Health Network, Laboratory Medicine Program, Toronto, ON, M5G 2C4, Canada; cg University of Helsinki, Department of Obstetrics and Gynecology, Helsinki University Hospital, Helsinki, 00290, Finland; ch Memorial Sloan Kettering Cancer Center, Clinical Genetics Research Lab, Department of Cancer Biology and Genetics, New York, NY, 10065, USA; ci Memorial Sloan Kettering Cancer Center, Clinical Genetics Service, Department of Medicine, New York, NY, 10065, USA; cj Centro de Investigación en Red de Enfermedades Raras (CIBERER), Madrid, 28029, Spain; ck Seoul National University College of Medicine, Integrated Major in Innovative Medical Science, Seoul, 03080, South Korea; cl American Cancer Society, Department of Population Science, Atlanta, GA, 30303, USA; cm University of Oulu, Laboratory of Cancer Genetics and Tumor Biology, Cancer and Translational Medicine Research Unit, Biocenter Oulu, Oulu, 90570, Finland; cn Northern Finland Laboratory Centre Oulu, Laboratory of Cancer Genetics and Tumor Biology, Oulu, 90570, Finland; co Fondazione IRCCS Istituto Nazionale dei Tumori (INT), Unit of Molecular Bases of Genetic Risk and Genetic Testing, Department of Research, Milan, 20133, Italy; cp Carmel Medical Center and Technion Faculty of Medicine, Clalit National Cancer Control Center, Haifa, 35254, Israel; cq Hospital Universitario Puerta de Hierro, Medical Oncology Department, Madrid, 28222, Spain; cr University Hospital of Larissa, Department of Oncology, Larissa, 411 10, Greece; cs King's College London, School of Cancer & Pharmaceutical Sciences, Comprehensive Cancer Centre, Guy's Campus, London, UK; ct University Hospital and German Cancer Research Center, National Center for Tumor Diseases, Heidelberg, 69120, Germany; cu Academia Sinica, Institute of Biomedical Sciences, Taipei, 115, Taiwan; cv China Medical University, School of Public Health, Taichung, Taiwan; cw University Hospital Heidelberg, Department of Pathology, Institute of Pathology, Heidelberg, 69120, Germany; cx University of Auckland, Auckland, New Zealand; cy Cancer Research Malaysia, Breast Cancer Research Programme, Subang Jaya, Selangor, 47500, Malaysia; cz Pontificia Universidad Javeriana, Institute of Human Genetics, Bogota, 110231, Colombia; da Mayo Clinic, Department of Quantitative Health Sciences, Division of Epidemiology, Rochester, MN, 55905, USA; db Erasmus University Medical Center, Department of Pathology, Rotterdam, 3015 CN, the Netherlands; dc The Netherlands Cancer Institute – Antoni van Leeuwenhoek Hospital, Division of Psychosocial Research and Epidemiology, Amsterdam, 1066 CX, the Netherlands

**Keywords:** Prognosis, PREDICT Breast, breast cancer, Progesterone receptor

## Abstract

**Background:**

Predict Breast (www.predict.nhs.uk) is an online prognostication and treatment benefit tool for early invasive breast cancer. The aim of this study was to incorporate the prognostic effect of progesterone receptor (PR) status into a new version of PREDICT and to compare its performance to the current version (2.2).

**Method:**

The prognostic effect of PR status was based on the analysis of data from 45,088 European patients with breast cancer from 49 studies in the Breast Cancer Association Consortium. Cox proportional hazard models were used to estimate the hazard ratio for PR status. Data from a New Zealand study of 11,365 patients with early invasive breast cancer were used for external validation. Model calibration and discrimination were used to test the model performance.

**Results:**

Having a PR-positive tumour was associated with a 23% and 28% lower risk of dying from breast cancer for women with oestrogen receptor (ER)-negative and ER-positive breast cancer, respectively. The area under the ROC curve increased with the addition of PR status from 0.807 to 0.809 for patients with ER-negative tumours (*p* = 0.023) and from 0.898 to 0.902 for patients with ER-positive tumours (*p* = 2.3 × 10^−6^) in the New Zealand cohort. Model calibration was modest with 940 observed deaths compared to 1151 predicted.

**Conclusion:**

The inclusion of the prognostic effect of PR status to PREDICT Breast has led to an improvement of model performance and more accurate absolute treatment benefit predictions for individual patients. Further studies should determine whether the baseline hazard function requires recalibration.

## Highlights

• PR) status is associated with a better prognosis after adjusting for other factors.
• PREDICT with the inclusion of PR was assessed by calibration and discrimination.
• addition of PR status improved model performance in terms of discrimination.
• Calibration in an external dataset showed over-estimation of breast cancer mortality.

## 1 Introduction

Accurate predictions of individualised survival estimates and benefits of adjuvant therapy following surgery are essential for clinical decision-making for patients with early invasive breast cancer. PREDICT Breast (www.breast.predict.nhs.uk) is an online prognostication and treatment benefit tool to aid clinical decision-making for adjuvant therapy after surgery for patients with early invasive breast cancer [1]. The model uses information about age at diagnosis and tumour characteristics to predict 5-, 10- and 15-year mortality and the benefit of treatment of adjuvant cytotoxic chemotherapy, hormone therapy, trastuzumab and/or bisphosphonate therapy. The clinico-pathological factors used in the current version (v2.2) are tumour size, tumour grade, number of positive lymph nodes, oestrogen receptor (ER) status, human epidermal growth factor receptor 2 (HER2) status, KI67 status and mode of detection [[1], [2], [3]]. PREDICT Breast was developed using cancer registry data from 5694 women diagnosed in East Anglia, United Kingdom, between 1999 and 2003 [4]. Separate breast cancer-specific mortality models were derived for ER-negative tumours and ER-positive tumours. The survival for patients with breast cancer is estimated by the hazard ratios of the risk factors in combination with the baseline survival function derived from a Cox proportional hazards regression model. It is possible to include additional prognostic factors into the model, even if data on those factors were not available in the data used to derive the model, by applying the external estimates of prognostic effects to the baseline hazard function. This approach was used to incorporate HER2 status and KI67 status, which led to an improvement in predictive performance [2,3].

Progesterone receptor (PR) status is a biomarker that has been shown to be prognostic in early invasive breast cancer in a large number of studies [[5], [6], [7], [8], [9], [10], [11]]. It is usually assessed by immunohistochemistry and, in combination with ER status and HER2 status, can be used to classify the breast carcinoma subtype [7]. Furthermore, the expression levels of PR predict clinical outcomes and the beneficial effect of adjuvant hormonal treatments [6,[8], [9], [10]]. Thus, the addition of PR status to the PREDICT Breast model has the potential to improve the discrimination of the model and improve its clinical utility.

We had two specific aims. The first was to obtain estimates of the relative hazard for breast cancer-specific mortality associated with PR status after adjusting for the prognostic factors included in PREDICT Breast v2.2. The second was to incorporate this hazard ratio estimate into the PREDICT Breast model and compare the performance of the new model against the current model (PREDICT Breast version 2.2).

## 2 Methods

### 2.1 Prognostic effect of biomarker PR status

We evaluated the prognostic effect of PR status using data on patients with breast cancer of European ancestries collected by 49 studies in the Breast Cancer Association Consortium (BCAC) (Supplementary Table S1). All contributing studies were approved by the relevant research ethics committee. Data for women diagnosed with early invasive breast cancer between 1990 and 2017 with complete information on the primary clinico-pathological factors used in the current version of PREDICT v2.2 – tumour size, tumour grade, number of positive lymph nodes, ER status, PR status – were included in the analyses. HER2 status was also available for most patients and could be included as PREDICT allows for missing HER2 data. The mode of detection was missing for 85% of the cases: we assumed that patients aged younger than 50 years or older than 70 years at diagnosis had been clinically detected and mean imputation was used for the remaining missing data. Cases with the following characteristics were excluded: aged younger than 25 or older than 85 at diagnosis, tumour diameter over 20 cm, more than 20 positive lymph nodes. PR status was available for 45,088 patients (13,706 PR-negative tumours and 31,382 PR-positive tumours) (Table 1). Data on ER, HER2 and PR status were collected separately by each study. For some studies, the data were from clinical records – and the definition of positivity may have varied from hospital to hospital. Other studies collected pathology material and carried out immunohistochemistry for these markers as part of the research. Different scoring systems and different definitions of positivity were used by different studies. Vital status and cause of death were obtained from the hospital medical records or the cancer registry or via linkage to death notifications.

**Table 1:** Patient characteristics for the BCAC studies with European patients with breast cancer (*n* = 45,088) and the New Zealand validation cohort (*n* = 11,365).

	BCAC European ancestries		New Zealand cohort	
	N	Mean (sd), unless stated otherwise	N	Mean (sd), unless stated otherwise
**Age, years**	45,088	57.1 (11.9)	11,365	57.1 (12.2)
**Follow-up time, years**	45,088	8.1 (5.0)	11,365	5.3 (3.6)
**Tumour size, cm**	45,088	2.1 (1.5)	11,365	2.3 (1.7)
**Tumour grade, n (%)**				
Grade 1	45,088	8776 (19.5)	11,365	2841 (25.0)
Grade 2		21,945 (48.7)		5312 (46.7)
Grade 3		14,367 (31.9)		3212 (28.3)
**ER/PR status, n(%)**				
ER−/PR−	45,088	7474 (16.6)	11,365	2026 (17.8)
ER−/PR+		1187 (2.6)		168 (1.5)
ER+/PR−		6232 (13.8)		1583 (13.9)
ER+/PR+		30,195 (67.0)		7588 (66.8)
**HER2 status, n (%)**				
Negative	32,328	27,108 (83.9)	9213	7774 (84.4)
Positive		5220 (16.1)		1439 (15.6)
**No. of positive lymph nodes**	45,088	1.2 (2.7)	11,365	1.7 (3.4)
**Mode of detection, n (%)**				
Clinically detected	45,088	21,639 (48.0)	11,365	6516 (57.3)
Screen detected		2433 (5.4)		4849 (42.7)
Missing		21,016 (46.6)		
**Chemotherapy, n (%)**				
No	36,991	20,157 (54.5)	11,365	7391 (65.0)
Yes		16,834 (45.5)		3974 (35.0)
**Hormone therapy, n (%)**				
No	35,486	10,724 (30.2)	11,365	4340 (38.2)
Yes		24,762 (69.8)		7025 (61.8)
**Radiotherapy, n(%)**				
No	32,166	8360 (26.0)		
Yes		23,806 (74.0)		
**Trastuzumab, n(%)**				
No	22,529	20,997 (93.2)		
Yes		1532 (6.8)		
**Number of deaths, n(%)**	45,081	6974 (15.5)	11,365	1609 (14.2)
**Causes of death, n(%)**				
Breast cancer	5925	3531 (59.6)	1609	940 (58.2)
Other causes		2394 (40.4)		568 (35.3)
Unknown causes				101 (6.3)

We estimated the hazard ratio for PR-positive tumours compared with PR-negative tumours using a Cox proportional hazards model for time to death from breast cancer stratified by study and adjusted for the PREDICT Breast v2.2 prognostic score. The PREDICT Breast v2.2 prognostic score (a log hazard ratio) was calculated for each case according to the formula reported in Candido dos Reis *et al.* (Table 1) [1]. Follow-up time was defined as the time from diagnosis to last follow-up or death from breast cancer or 15 years after diagnosis, whichever came first. In order to account for prevalent cases, time at risk started at the study entry (left truncation). This provides an unbiased estimate of the hazard ratio [12]. Separate models were derived for ER-negative breast cancer cases and ER-positive breast cancer cases.

### 2.2 Incorporation of PR status into PREDICT breast

The absolute risk of breast cancer-specific mortality is estimated in PREDICT Breast by applying the prognostic score to an estimate of the baseline hazard that was developed using a cohort of breast cancer cases with unknown information on PR status. Thus, the underlying baseline hazard represents breast cancer cases with an average PR status. The estimates of the prognostic effects of PR status for ER-negative tumours and ER-positive tumours were, therefore, rescaled to give an average hazard ratio of unity using a prevalence of PR positivity of 14% in ER-negative cases and 83% in ER-positive tumours.

### 2.3 Validation study population

Data from a New Zealand population-based cancer registry were used for model validation [13]. Data were available on 11,365 patients with early invasive breast cancer (2194 ER-negative and 9171 ER-positive) diagnosed between 2000 and 2014 after the exclusion of cases with metastasis at diagnosis (639), those younger than 25 or older than 85 years old (524), tumour diameter larger than 20 cm (5), more than 20 positive lymph nodes (232), inconsistent follow-up time information (2) and those that did not undergo primary surgery (938).

Information on adjuvant systemic cancer treatments, chemotherapy and hormone therapy were also recorded. The New Zealand cohort did not include information on specific chemotherapy regimes. To derive the prognostic score, we assumed that patients who underwent chemotherapy before 2010 were treated with anthracycline-based regimen, and for those treated after this time, we assumed a taxane-based regimen. This is based on data for the most commonly used regimen in New Zealand (Mark Elwood personal communication). In addition, information on the use of trastuzumab was not collected during follow-up. We assume that patients with a positive HER2 tumour and that were diagnosed after 2010, underwent trastuzumab treatment.

The dates and causes of death were extracted from the hospital records and from mortality records until 31st December 2014 and all patients were censored after this date. The primary end-point was breast cancer-speciﬁc survival. The expected survival probability for each patient was based on a follow-up time that was different for each patient up to a maximum of 15 years. For patients who survived, follow-up was from the date of diagnosis until the date of last follow-up. For patients who died, potential follow-up time was calculated as if the patient had survived to the end of the study, which is from the date of diagnosis until 31st December 2014.

For each patient, their breast cancer risk predictions were estimated using the two models; PREDICT version 2.2 and PREDICT version 2.2 with the inclusion of PR status (v2.3). Model calibration was performed to investigate the accuracy of the mortality estimates predicted by each model compared to the observed mortality rate. Additionally, a Chi-square test was used as a goodness-of-ﬁt test in which the observed events were also compared with the number of predicted events (1 d.f.). Model discrimination was also evaluated through the calculation of the AUC (area under the receiver–operator–characteristic curve) for up to 15-year breast cancer mortality. The AUC was used to measure the accuracy of the classification of cases and non-cases for the two prediction models and to test for any beneficial effect of the addition of PR status to PREDICT Breast. The comparison of AUCs was done using the method of De Long *et al.* [14] implemented in the R package *pROC*. All analyses were conducted using R v4.1.2 in the R Studio environment.

## 3 Results

The 49 BCAC studies included 45,088 eligible European patients of whom 13,706 (30%) had PR-negative tumours and 31,382 (70%) had PR-positive tumours (Table 1). During follow-up, there were 6974 recorded deaths with approximately 11 breast cancer deaths per 1000 person-years. The patient characteristics of the New Zealand cohort were very similar to those in the studies of BCAC apart from the proportion of patients that underwent chemotherapy (35%), which was lower than that for BCAC (46%).

Initial analyses were restricted to patients of European ancestries. In univariate analyses, PR expression was associated with a better prognosis, with the magnitude of the effect being greater in ER-positive disease (Table 2). The effect of PR expression was attenuated after adjusting for other prognostic factors. We evaluated whether the effect of PR varied by age or HER2 status by including an interaction term in the multi-variable model. There was little evidence for the interaction in either age at diagnosis (*p* = 0.65 in ER-positive and *p* = 0.43 in ER-negative) or HER2 status (*p* = 0.36 in ER-positive and *p* = 0.91 in ER-negative).

**Table 2:** Hazard ratios (95% C.I.) for progesterone receptor (PR) status and other prognostic factors for breast cancer-specific mortality stratified by oestrogen receptor (ER) status and study derived from the BCAC data for European ancestries.

	ER-negative	*p*-value	ER-positive	*p*-value
	HR (95% C.I.)		HR (95% C.I.)	
*Univariable*				
PR+ v PR−	0.65 (0.54–0.80)	2.0 × 10^−5^	0.60 (0.55–0.67)	<10^−15^
*Multivariable with PREDICT prognostic index*^a^				
PR+ v PR−	0.77 (0.64–0.94)	0.009	0.72 (0.65–0.79)	3.7 × 10^−11^
*Multivariable with individual prognostic factors*				
PR+ v PR−	0.76 (0.60–0.98)	0.031	0.69 (0.62–0.78)	1.6 × 10^−9^
Age diagnosis (per 5 years)	1.04 (1.00–1.08)	0.028	1.03 (1.00–1.06)	0.030
Size (per cm)	1.17 (1.13–1.22)	<10^−15^	1.13 (1.10–1.16)	<10^−15^
Nodes (per positive node)	1.13 (1.11–1.14)	<10^−15^	1.12 (1.10–1.13)	<10^−15^
Grade				
2 versus 1	2.22 (1.20–4.10)	0.011	2.51 (2.04–3.08)	<10^−15^
3 versus 1	2.52 (1.38–4.62)	2.7 × 10^−3^	4.26 (3.44–5.28)	<10^−15^
Screen detected versus clinically detected	0.65 (0.45–0.93)	0.018	0.53 (0.43–0.65)	1.1 × 10^−9^
HER2+ v HER2−	0.96 (0.81–1.13)	0.603	1.10 (0.96–1.26)	0.163

a PRS coefficient constraint to be one.

We also assessed between-study heterogeneity and plotted the estimated beta coefficient of PR status per study adjusted for the prognostic index (Supplementary Fig. S1). There was no evidence of heterogeneity in the ER-negative model (*p* = 0.99) or in the ER-positive model (*p* = 0.26).

The visual examination of plots of log-cumulative hazard against log-time and the Schoenfeld residuals against time showed that there was no serious violation of the proportional hazards assumption (Supplementary Figs. S2 and S3). The hazard ratios for the other prognostic factors from the multivariable model that included each prognostic factor separately were slightly different to those in the PREDICT model. Of particular note is that in the BCAC dataset, a significant association was observed for the mode of detection in ER-negative disease. It has previously been reported to be associated only in ER-positive tumours.

In order to apply the PR hazard ratio to the PREDICT Breast baseline hazard, it needed to be rescaled such that the mean hazard ratio was unity with the purpose that the reference category for the hazard ratio is a hypothetical case with average PR status. The proportion of cases that are PR-positive used for rescaling was the average from the combined BCAC studies (14% for ER-negative and 83% for ER-positive cases). The rescaled hazard ratios were 1.03 for PR-negative/ER-negative, 0.80 for PR-positive/ER-negative, 1.30 for PR-negative/ER-positive and 0.94 for PR-positive/ER-positive. The hazard ratios for all the other prognostic variables and the baseline hazard function remained unchanged from PREDICT Breast v2.2.

**Fig. 1:**
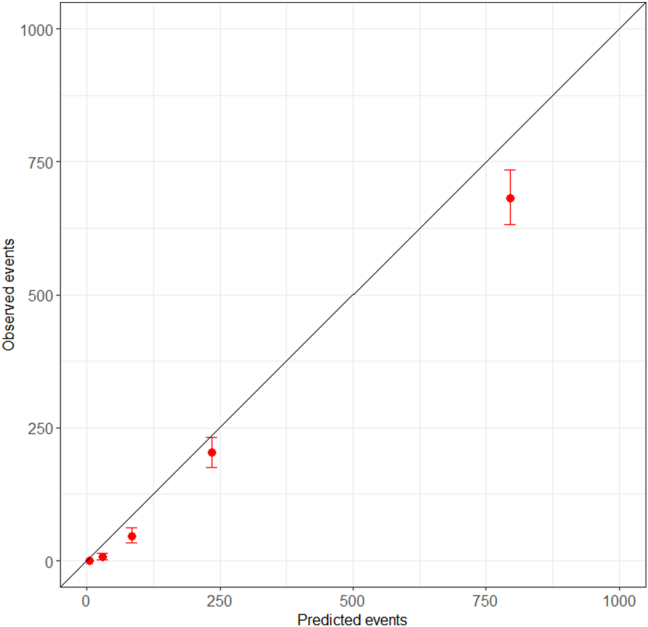
Calibration plot of observed outcomes at 15 years after diagnosis with 95% confidence intervals against 15-year predicted outcomes at by quintiles of the predicted value in the New Zealand cohort.

The performance of PREDICT Breast v2.2 with the addition of PR status was then evaluated in the independent New Zealand data set and compared with v2.2. The discrimination for up to 15-year breast cancer-specific mortality of PREDICT as measured by the AUC increased from 0.807 to 0.809 (*p* = 0.023) for patients with ER-negative breast cancer and from 0.898 to 0.902 (*p* = 2.3 × 10^−6^) for ER-positive cases (Table 3). The calibration of the model was modest, with 1151 breast cancer deaths predicted compared to 940 that were observed during a 15-year follow-up (goodness-of-fit Chi-squared test *p* = 5.0 × 10^−10^) (Table 4). Over-estimation was worse in European patients with ER-negative tumours (366 predicted compared with 281 observed, *p* = 8.9 × 10^−6^) than European patients with ER-positive tumours (442 predicted compared to 414 observed, *p* = 0.183). Across ethnicities, the model performs better in ER-positive cases in comparison to ER-negative cases. Fig. 1 shows the calibration of PREDICT Breast including PR status across the quintiles of predicted risk.

**Table 3:** The discrimination for up to 15-year breast cancer-specific mortality in the New Zealand validation cohort.

	C-index without PR status	C-index with PR status	*p*-value
**ER specific**			
ER-negative	0.807	0.809	0.023
ER-positive	0.898	0.902	2.3 × 10^−6^
**Ethnicity**			
Māori	0.901	0.901	0.983
Pacific	0.897	0.898	0.883
European	0.878	0.881	1.0 × 10^−6^
Other ethnicity	0.919	0.923	0.022
Overall	0.885	0.888	1.5 × 10^−7^

**Table 4:** Cumulative observed versus predicted breast cancer deaths at up to 15 years follow-up by ethnicity in the New Zealand cohort.

	Total number of breast cancer patients by ethnic group	Predicted breast cancer-specific mortality		Observed breast cancer-specific mortality
		Without PR status	With PR status	
**Number of deaths**				
Māori	1054	117	117	108
Pacific	666	90	90	70
European	8220	799	808	695
Other	1257	121	122	66
Missing	168	14	14	1
Total	11,365	1141	1151	940
**ER specific**				
ER−				
Māori	177	52	52	44
Pacific	153	43	44	31
European	1576	363	366	281
Other	258	58	58	35
Missing	30	7	8	1
Total	2194	523	528	392
ER+				
Māori	877	65	65	64
Pacific	513	47	46	39
European	6644	436	442	414
Other	999	63	64	31
Missing	138	6	6	0
Total	9171	617	623	548

The number of observed and predicted deaths from other causes and deaths from all causes in the New Zealand cohort are shown in Table 5, Table 6. Overall, PREDICT Breast with the inclusion of PR status shows to be well-calibrated in predicting non-breast-cancer-specific mortality with an over-estimation of 0.4% (670 predicted compared with 667 observed, *p* = 0.908). The model shows to be slightly over-estimating the number of non-breast cancer deaths in patients of European descent by 6.8% (546 predicted compared with 511 observed, *p* = 0.134), whilst in patients from Pacific origin they are slightly under-estimated (28 predicted compared with 32 observed, *p* = 0.450). While the model performs better in these ethnic groups, it performs worse in Māori patients (40 predicted compared with 88 observed, *p* = 3.2 × 10^−14^). Both models (PREDICT Breast versus PREDICT Breast including PR status) show to over-estimate the all-cause mortality by approximately 13%, regardless of ER status. Similar to the other-cause mortality results, the models show to over-estimate the number of predicted all-cause deaths in most ethnic groups. However, there is an under-estimation of all-cause deaths in patients of Māori descent.

**Table 5:** Cumulative observed versus predicted other-cause/non-breast cancer deaths at up to 15 years follow-up by ethnicity in the New Zealand cohort.

	Total number of breast cancer patients by ethnic group	Other-cause/non-breast cancer-specific mortality		Observed other-cause mortality
		Without PR status	With PR status	
**Number of deaths**				
Māori	1054	40	40	88
Pacific	666	27	28	32
European	8220	547	546	511
Other	1257	47	47	36
Missing	168	9	9	0
Total	11,365	671	670	667
**ER specific**				
ER−				
Māori	177	7	7	16
Pacific	153	6	6	6
European	1576	101	101	100
Other	258	9	9	8
Missing	30	2	2	0
Total	2194	125	125	130
ER+				
Māori	877	34	34	72
Pacific	513	21	21	26
European	6644	446	446	411
Other	999	38	38	28
Missing	138	7	7	0
Total	9171	546	546	537

**Table 6:** Cumulative observed versus predicted all-cause deaths at up to 15 years follow-up by ethnicity in the New Zealand cohort.

	Total number of breast cancer patients by ethnic group	All-cause mortality		Observed all-cause mortality
		Without PR status	With PR status	
**Number of deaths**				
Māori	1054	157	157	196
Pacific	666	118	118	102
European	8220	1346	1355	1206
Other	1257	168	169	102
Missing	168	23	23	1
Total	11,365	1811	1821	1607
**ER specific**				
ER−				
Māori	177	59	59	60
Pacific	153	50	50	37
European	1576	464	467	381
Other	258	67	67	43
Missing	30	9	9	1
Total	2194	648	652	522
ER+				
Māori	877	98	98	136
Pacific	513	68	67	65
European	6644	882	888	825
Other	999	101	102	59
Missing	138	14	13	0
Total	9171	1163	1169	1085

We then carried out a sensitivity analysis using the alternative assumptions for chemotherapy and trastuzumab treatment. Table S2 shows the predicted breast cancer deaths with the assumption that patients who underwent chemotherapy were treated with anthracycline-based regimen (second-generation regimen). Table S3 shows the predicted breast cancer deaths with the assumptions that all patients with HER2-positive tumours were treated with trastuzumab, and patients who underwent chemotherapy and were diagnosed before 2010 were treated with anthracycline-based regimen and for those diagnosed after this time were treated with a taxane-based regimen. The model appears to be miscalibrated and results show that the calibration is sensitive to the treatment assumptions made prior to the analyses.

In order to determine the clinical impact of the small improvement in discrimination, we estimated the reclassification of risk for PREDICT v2.2 + PR compared to PREDICT v 2.2 based on classifying cases from the New Zealand cohort into three categories of breast cancer-specific mortality at ten years, less than 15%, 15% to less than 20% and 20% or greater. These thresholds are approximately equivalent to the thresholds for the absolute risk reduction of chemotherapy of 3% and 5% used by the Cambridge Breast Unit Multidisciplinary Team for clinical decision-making [15]. Table 7 shows that in total 4.2% of cases changed risk category, of which 2.4% changed from a lower risk category to a higher risk category.

**Table 7:** Reclassification of predicted breast cancer-specific mortality following the inclusion of PR status into PREDICT Breast.

	Predicted breast cancer-specific mortality	PREDICT Breast (including PR status)		
		[0.00–0.15)	[0.15–0.20)	[0.20–1.00]
**PREDICT Breast**	**[0.00–0.15)**	7191 (63.3%)	137 (1.2%)	0 (0%)
	**[0.15–0.20)**	115 (1.0%)	859 (7.6%)	138 (1.2%)
	**[0.20–1.00]**	0 (0%)	92 (0.8%)	2833 (24.9%)

## 4 Discussion

The primary aim of this study was to estimate the prognostic effect – as the relative hazard – of PR expression in breast cancer after adjusting for the other prognostic factors incorporated in the PREDICT Breast prognostic tool. Importantly, the effects of other prognostic factors were constrained to the same effect sizes as used in the PREDICT Breast model. This enabled us to incorporate progesterone expression into PREDICT Breast by applying the relative hazard to the baseline hazard which is specified in the PREDICT Breast model. The BCAC data set on which this analysis was based is large, with over 45,000 cases of European ancestries from 49 separate studies from around the world and over 3500 deaths from breast cancer during follow-up. In addition to the large sample size, the heterogeneity inherent in combining data from multiple studies is strength as the findings should be robust and widely generalisable. While a large number of cases of south Asian ancestries were also available from the BCAC data set, there were a small number of breast cancer deaths during the follow-up and impact of ancestry on the association between PR expression and prognosis could not be reliably assessed.

The heterogeneity of study design and conduct is also reflected in the measurement of the prognostic factors included in the analyses. In particular, different studies used different data sources to determine ER, HER2 and PR status including clinical records and research data. Consequently, different studies used slightly different definitions to classify ER, HER2 and PR status and these data could not be fully harmonised across studies. Any measurement error resulting from this is likely to have biased the association of PR status with survival towards the null but any such bias is expected to be small.

Our results are broadly similar to the extensive published data [[5], [6], [7], [8], [9], [10], [11],^16^,^17^] and show that patients with a positive PR tumour have a better survival than patients with a PR-negative tumour regardless of their ER status. There was little difference in the relative hazard estimates after adjusting for a prognostic index constrained to the effect size used in the PREDICT Breast model or in full, multi-variable model that allowed the hazard ratios for the other prognostic factors to fit the data. Previous reports have shown that the prognostic effect of PR status varies with age at diagnosis with a bigger effect being observed in younger patients [16,17], particularly during the first five years of follow in one of the studies [16]. However, we found little evidence for a difference in the effect with age.

We used the relative hazard estimates to incorporate progesterone receptor expression into the PREDICT Breast model and compared the performance of the modified model with that of the current version of PREDICT Breast as used in the online web tool (v2.2). This was done using a completely independent data set from New Zealand. The addition of a single prognostic factor to a multi-variable prediction model would not be expected to improve the performance of the model substantially. Nevertheless, the addition of PR status resulted in a small, but statistically significant improvement in the discrimination of PREDICT Breast compared with the current version. Similarly, the small proportion of patients being reclassified when using clinically relevant categories of risk that was observed was as would be expected. The calibration of the modified version of PREDICT Breast would not be expected to change much as calibration is primarily dependent on the baseline hazard which was the same in the modified and current models and then depends on the assumption about the proportion of cases that are PR-positive used to rescale the hazard ratios as described in the methods. The calibration of the modified models in an independent data set was modest with the number of breast cancer deaths in the New Zealand cohort being over-estimated by 22%. This was, as expected, similar – albeit slightly worse – to the calibration of the current model. The miscalibration was similar for all ancestries and was worse in patients with ER-negative. PREDICT Breast has previously been shown to be well-calibrated in cases series from the UK, Canada, the Netherlands and Malaysia, and the reasons for the poorer performance in the New Zealand data set are not clear. One possible explanation is that the baseline hazard for PREDICT is based on a cohort of patients from the UK diagnosed from 1999 to 2004 whereas the New Zealand cohort was diagnosed from 2000 to 2014. There have been improvements in prognosis over time and so some over-estimation of deaths is expected. This is supported by the observation that there is an improvement in the calibration of PREDICT Breast including PR status when performing analysis on patients diagnosed between 2000 and 2004, with an over-estimation in breast cancer deaths of 7.7% in all patients and 3.6% in European patients, compared to 22.4% and 16.3% for patients diagnosed between 2000 and 2014. Some of these improvements are the result of the introduction of newer therapies such as bisphosphonates, increased the duration of hormone therapies and improvements in the management of disease at the time of relapse. However, information on these therapies was not available for the validation data and so could not be accounted for in the analyses. A simple country-specific recalibration of the baseline hazard function or a re-estimation of the baseline hazard using more contemporaneous data would improve the model performance.

The expression of biomarkers such as ER, HER2 and PR is continuous but then dichotomised based on a threshold for use in clinical practice. For ER and HER2 status, this is primarily done to facilitate decision-making for specific adjuvant therapies. There is good evidence that the prognostic effect of these biomarkers varies with the level of expression [[18], [19], [20]] and the inclusion of a multi-category ordinal scale or a continuous measure of expression in the model has the potential to improve model performance.

In conclusion, the incorporation of the prognostic effect of PR status into PREDICT Breast has resulted in a small, statistically significant improvement in discrimination with some reclassification in clinically relevant risk thresholds. On the other hand, the calibration of the modified PREDICT model in an independent data set was slightly poorer. The improvement in discrimination is likely to be generalisable across diverse case cohorts as it is primarily dependent on the magnitude of the hazard ratio associated with progesterone receptor status which is likely to be robustly estimated. In contrast, calibration is dependent on the baseline hazard which may vary across different populations and time periods as well as the distribution of the biomarker in different populations. Thus, progesterone receptor expression will be included into a new version of PREDICT Breast (v2.3) based on the improvement in discrimination and the reclassification. Further studies should investigate the potential improvement that recalibrating the baseline hazard function could have on country-specific model performance.

## Funding

BCAC is funded by the European Union's Horizon 2020 Research and Innovation Programme (grant numbers 634935 and 633784 for BRIDGES and B-CAST respectively), and the PERSPECTIVE I&I project, funded by the 10.13039/501100000023Government of Canada through Genome Canada and the 10.13039/501100000024Canadian Institutes of Health Research, the Ministère de l'Économie et de l'Innovation du Québec through Genome Québec, the 10.13039/100016328Quebec Breast Cancer Foundation. The EU Horizon 2020 Research and Innovation Programme funding source had no role in study design, data collection, data analysis, data interpretation or writing of the report. Additional funding for BCAC is provided via the Confluence project which is funded with intramural funds from the National Cancer Institute Intramural Research Program, 10.13039/100000002National Institutes of Health.

The ABCS study was supported by the Dutch Cancer Society [grants NKI 2007-3839; 2009 4363]. The Australian Breast Cancer Tissue Bank (ABCTB) was supported by the 10.13039/501100000925National Health and Medical Research Council of Australia, The Cancer Institute NSW and the 10.13039/501100001026National Breast Cancer Foundation. The work of the BBCC was partly funded by ELAN-Fond of the University Hospital of Erlangen. The BCINIS study is supported in part by the 10.13039/100001006Breast Cancer Research Foundation (BCRF). For BIGGS, ES is supported by NIHR Comprehensive Biomedical Research Centre, 10.13039/501100004941Guy's and St Thomas' NHS Foundation Trust in partnership with 10.13039/501100000764King's College London, United Kingdom. IT is supported by the Oxford Biomedical Research Centre. The BREast Oncology GAlician Network (BREOGAN) is funded by Acción Estratégica de Salud del Instituto de Salud Carlos III FIS PI12/02125/Cofinanciado and 10.13039/501100002924FEDER PI17/00918/Cofinanciado FEDER; Acción Estratégica de Salud del Instituto de Salud Carlos III FIS Intrasalud (PI13/01136); Programa Grupos Emergentes, Cancer Genetics Unit, Instituto de Investigacion Biomedica Galicia Sur. Xerencia de Xestion Integrada de Vigo-SERGAS, Instituto de Salud Carlos III, Spain; Grant 10CSA012E, Consellería de Industria Programa Sectorial de Investigación Aplicada, PEME I + D e I + D Suma del Plan Gallego de Investigación, Desarrollo e Innovación Tecnológica de la Consellería de Industria de la Xunta de Galicia, Spain; Grant EC11-192. Fomento de la Investigación Clínica Independiente, Ministerio de Sanidad, Servicios Sociales e Igualdad, Spain; and Grant FEDER-Innterconecta. Ministerio de Economia y Competitividad, Xunta de Galicia, Spain. The BSUCH study was supported by the Dietmar-Hopp Foundation, the Helmholtz Society and the German Cancer Research Center (DKFZ). CCGP is supported by funding from the 10.13039/501100004429University of Crete. The CGPS was supported by the Chief Physician Johan Boserup and Lise Boserup Fund, the 10.13039/100008392Danish Medical Research Council, and Herlev and Gentofte Hospital. The CNIO-BCS was supported by the 10.13039/501100004587Instituto de Salud Carlos III, the Red Temática de Investigación Cooperativa en Cáncer and grants from the Asociación Española Contra el Cáncer and the Fondo de Investigación Sanitario (PI11/00923 and PI12/00070). COLBCCC is supported by the 10.13039/100008658German Cancer Research Center, Heidelberg, Germany. Diana Torres was in part supported by a postdoctoral fellowship from the 10.13039/100005156Alexander von Humboldt Foundation. The 10.13039/100000048American Cancer Society funds the creation, maintenance, and updating of the CPS-II cohort. The University of Westminster curates the DietCompLyf database funded by 10.13039/100013129Against Breast Cancer Registered Charity No. 1121258 and the NCRN. FHRISK and PROCAS are funded from NIHR grant PGfAR 0707-10031. DGE, AH and WGN are supported by the 10.13039/100014653NIHR Manchester Biomedical Research Centre (IS-BRC-1215-20007). The HABCS study was supported by the Claudia von Schilling Foundation for Breast Cancer Research, by the Lower Saxonian Cancer Society, and by the Rudolf Bartling Foundation. The HEBCS was financially supported by the Helsinki University Hospital Research Fund, the Finnish Cancer Society, and the 10.13039/501100006306Sigrid Jusélius Foundation. The HERPACC was supported by MEXT Kakenhi (No. 170150181 and 26253041) from the Ministry of Education, Science, Sports, Culture and Technology of Japan, by a Grant-in-Aid for the Third Term Comprehensive 10-Year Strategy for Cancer Control from 10.13039/501100003478Ministry of Health, Labour and Welfare of Japan, by Health and Labour Sciences Research Grants for Research on Applying Health Technology from 10.13039/501100003478Ministry of Health, Labour and Welfare of Japan, by National Cancer Center Research and Development Fund, and “Practical Research for Innovative Cancer Control (15ck0106177h0001 and 20ck0106553)” from 10.13039/100009619Japan Agency for Medical Research and Development, AMED, and Cancer Bio Bank Aichi. Financial support for KARBAC was provided through the regional agreement on medical training and clinical research (ALF) between Stockholm County Council and 10.13039/501100004047Karolinska Institutet, the 10.13039/501100002794Swedish Cancer Society, The Gustav V Jubilee foundation and Bert von Kantzows foundation. The KARMA study was supported by Märit and Hans Rausings Initiative Against Breast Cancer. kConFab is supported by a grant from the 10.13039/501100001026National Breast Cancer Foundation, and previously by the 10.13039/501100000925National Health and Medical Research Council, the Queensland Cancer Fund, the Cancer Councils of New South Wales, Victoria, Tasmania and South Australia, and the Cancer Foundation of Western Australia. Financial support for the AOCS was provided by the United States Army Medical Research and Materiel Command [DAMD17-01-1-0729], 10.13039/501100000951Cancer Council Victoria, Queensland Cancer Fund, 10.13039/501100001102Cancer Council New South Wales, 10.13039/501100000950Cancer Council South Australia, The Cancer Foundation of Western Australia, 10.13039/501100001169Cancer Council Tasmania and the 10.13039/501100000925National Health and Medical Research Council of Australia (NHMRC; 400413, 400281, 199600). G.C.T. and P.W. are supported by the 10.13039/501100000925NHMRC. RB was a Cancer Institute NSW Clinical Research Fellow. The KOHBRA study was partially supported by a grant from the Korea Health Technology R&D Project through the 10.13039/501100003710Korea Health Industry Development Institute, and the National R&D Program for Cancer Control, Ministry of Health & Welfare, Republic of Korea (HI16C1127; 1020350; 1420190). LMBC is supported by the ‘10.13039/501100005026Stichting Tegen Kanker’. DL is supported by the 10.13039/501100003130FWO. The MARIE study was supported by the Deutsche Krebshilfe e.V. [70-2892-BR I, 106332, 108253, 108419, 110826, 110828], the 10.13039/100018515Hamburg Cancer Society, the 10.13039/100008658German Cancer Research Center and the 10.13039/501100002347Federal Ministry of Education and Research Germany [01KH0402]. MBCSG is supported by grants from the 10.13039/501100005010Italian Association for Cancer Research. The MCBCS was supported by the NIH grants R35CA253187, R01CA192393, R01CA116167, R01CA176785 a NIH Specialised Program of Research Excellence (SPORE) in Breast Cancer [P50CA116201], and the 10.13039/100001006Breast Cancer Research Foundation. The Melbourne Collaborative Cohort Study (MCCS) cohort recruitment was funded by VicHealth and Cancer Council Victoria. The MCCS was further augmented by Australian National Health and Medical Research Council grants 209057, 396414 and 1074383 and by infrastructure provided by 10.13039/501100000951Cancer Council Victoria. Cases and their vital status were ascertained through the Victorian Cancer Registry and the Australian Institute of Health and Welfare, including the National Death Index and the Australian Cancer Database. The MEC was supported by NIH grants CA63464, CA54281, CA098758, CA132839 and CA164973. The MMHS study was supported by NIH grants CA97396, CA128931, CA116201, CA140286 and CA177150. MSKCC is supported by grants from the 10.13039/100001006Breast Cancer Research Foundation and Robert and Kate Niehaus Clinical Cancer Genetics Initiative. The work of MTLGEBCS was supported by the 10.13039/100016328Quebec Breast Cancer Foundation, the Canadian Institutes of Health Research for the “CIHR Team in Familial Risks of Breast Cancer” program – grant # CRN-87521 and the 10.13039/100011100Ministry of Economic Development, Innovation and Export Trade – grant # PSR-SIIRI-701. MYBRCA is funded by research grants from the 10.13039/100010269Wellcome Trust (v203477/Z/16/Z), the Malaysian Ministry of Higher Education (UM.C/HlR/MOHE/06) and Cancer Research Malaysia. The NBCS has received funding from the K.G. Jebsen Centre for Breast Cancer Research; the 10.13039/501100005416Research Council of Norway grant 193387/V50 (to A-L Børresen-Dale and V.N. Kristensen) and grant 193387/H10 (to A-L Børresen-Dale and V.N. Kristensen), South Eastern Norway Health Authority (grant 39346 to A-L Børresen-Dale) and the 10.13039/100008730Norwegian Cancer Society (to A-L Børresen-Dale and V.N. Kristensen). The Northern California Breast Cancer Family Registry (NC-BCFR) and Ontario Familial Breast Cancer Registry (OFBCR) were supported by grant U01CA164920 from the USA National Cancer Institute of the National Institutes of Health. The content of this manuscript does not necessarily reflect the views or policies of the National Cancer Institute or any of the collaborating centers in the Breast Cancer Family Registry (BCFR), nor does mention of trade names, commercial products or organisations imply endorsement by the USA Government or the BCFR. The OBCS was supported by research grants from the Finnish Cancer Foundation, the 10.13039/501100002341Academy of Finland (grant number 250083, 122715 and Center of Excellence grant number 251314), the Finnish Cancer Foundation, the 10.13039/501100006306Sigrid Jusélius Foundation, the 10.13039/501100006196University of Oulu, the University of Oulu Support Foundation and the special Governmental EVO funds for Oulu University Hospital-based research activities. The ORIGO study was supported by the Dutch Cancer Society (RUL 1997-1505) and the Biobanking and Biomolecular Resources Research Infrastructure (BBMRI-NL CP16). The PBCS was funded by Intramural Research Funds of the National Cancer Institute, Department of Health and Human Services, USA. The POSH study is funded by 10.13039/501100000289Cancer Research UK (grants C1275/A11699, C1275/C22524, C1275/A19187, C1275/A15956) and 10.13039/501100000301Breast Cancer Campaign 2010PR62, 2013PR044. The RBCS was funded by the Dutch Cancer Society (DDHK 2004–3124, DDHK 2009–4318). The SASBAC study was supported by funding from the 10.13039/501100001348Agency for Science, Technology and Research of Singapore (A∗STAR), the US National Institute of Health (NIH) and the 10.13039/100009634Susan G. Komen Breast Cancer Foundation. The SBCS was supported by Sheffield Experimental Cancer Medicine Centre and Breast Cancer Now Tissue Bank. SEARCH is funded by 10.13039/501100000289Cancer Research UK [C490/A10124, C490/A16561] and supported by the UK National Institute for Health Research Biomedical Research Centre at the 10.13039/501100000735University of Cambridge. The University of Cambridge has received salary support for PDPP from the NHS in the East of England through the Clinical Academic Reserve. SEBCS was supported by the 10.13039/100016229BRL program through the 10.13039/501100003725National Research Foundation of Korea funded by the 10.13039/501100004085Ministry of Education, Science and Technology (2012-0000347). SGBCC is funded by the 10.13039/501100001381National Research Foundation Singapore, NUS start-up Grant, 10.13039/501100011105National University Cancer Institute, Singapore Centre Grant, Breast Cancer Prevention Programme, Asian Breast Cancer Research Fund and the NMRC Clinician Scientist Award (SI Category). Population-based controls were from the Multi-Ethnic Cohort (MEC) funded by grants from the 10.13039/501100001350Ministry of Health -Singapore, 10.13039/501100001352National University of Singapore and 10.13039/501100011744National University Health System, Singapore. SKKDKFZS is supported by the 10.13039/100008658DKFZ. The SZBCS was supported by Grant PBZ_KBN_122/P05/2004 and the program of the Minister of Science and Higher Education under the name “Regional Initiative of Excellence” in 2019–2022 project number 002/RID/2018/19 amount of financing 12,000,000 PLN. The TWBCS is supported by the Taiwan Biobank project of the Institute of Biomedical Sciences, Academia Sinica, Taiwan. UBCS was supported by funding from 10.13039/100000054National Cancer Institute grant R01 CA163353 (to N.J. Camp) and the Women's Cancer Center at the 10.13039/100010638Huntsman Cancer Institute. Data collection for UBCS was supported by the Utah Population Database, 10.13039/100002799Intermountain Healthcare and the Utah Cancer Registry which is funded by the NCI's SEER Program (HHSN261201800016I), the US Centers for Disease Control and Prevention's National Program of Cancer Registries (NU58DP006320), with additional support from the 10.13039/100007747University of Utah and 10.13039/100010637Huntsman Cancer Foundation. The UCIBCS component of this research was supported by the NIH [CA58860, CA92044] and the Lon V Smith Foundation [LVS39420]. The UKBGS is funded by Breast Cancer Now and the Institute of Cancer Research (ICR), London. ICR acknowledges NHS funding to the NIHR Biomedical Research Centre.

## Author contributions

**Isabelle Grootes:** formal analysis, methodology, software, validation, writing – original draft, writing – review & editing, visualisation.

**Paul D.P. Pharoah:** conceptualisation, methodology, supervision, writing – review & editing, project administration.

**Renske Keeman and Manjeet K. Bolla:** investigation, data curation.

**Renske Keeman, Fiona M Blows, Roger L. Milne, Graham G. Giles, Anthony J. Swerdlow, Peter A. Fasching, Mustapha Abubakar, Irene L. Andrulis, Hoda Anton-Culver, Matthias W. Beckmann, Carl Blomqvist, Stig E. Bojesen, Manjeet K. Bolla, Bernardo Bonanni, Ignacio Briceno, Barbara Burwinkel, Nicola J. Camp, Jose E. Castelao, Ji-Yeob Choi, Christine L. Clarke, Fergus J. Couch, Angela Cox, Simon S. Cross, Kamila Czene, Peter Devilee, Thilo Dörk, Alison M. Dunning, Miriam Dwek, Douglas F. Easton, Diana M. Eccles, Mikael Eriksson, Kristina Ernst, D. Gareth Evans, Jonine D. Figueroa, Visnja Fink, Giuseppe Floris, Stephen Fox, Marike Gabrielson, Manuela Gago-Dominguez, José A. García-Sáenz, Anna González-Neira, Lothar Haeberle, Christopher A. Haiman, Per Hall, Ute Hamann, Elaine F. Harkness, Mikael Hartman, Alexander Hein, Maartje J. Hooning, Ming-Feng Hou, Sacha J. Howell, ABCTB Investigators, kConFab Investigators, Hidemi Ito, Anna Jakubowska, Wolfgang Janni, Esther M. John, Audrey Jung, Daehee Kang, Vessela N. Kristensen, Ava Kwong, Diether Lambrechts, Jingmei Li, Jan Lubiński, Mehdi Manoochehri, Sara Margolin, Keitaro Matsuo, Nur Aishah Mohd Taib, Anna Marie Mulligan, Heli Nevanlinna, William G. Newman, Kenneth Offit, Ana Osorio, Sue K. Park, Tjoung-Won Park-Simon, Alpa V. Patel, Nadege Presneau, Katri Pylkäs, Brigitte Rack, Paolo Radice, Gad Rennert, Atocha Romero, Emmanouil Saloustros, Elinor J. Sawyer, Andreas Schneeweiss, Fabienne Schochter, Minouk J. Schoemaker, Chen-Yang Shen, Rana Shibli, Peter Sinn, William J. Tapper, Essa Tawfiq, Soo Hwang Teo, Lauren R. Teras, Diana Torres, Celine M. Vachon, Carolien H.*M. van* Deurzen, Camilla Wendt, Justin A. Williams, Robert Winqvist, Mark Elwood, Marjanka K. Schmidt, Montserrat García-Closas:** investigation.

**Fiona M. Blows:** resources, data curation.

**Montserrat García-Closas and Marjanka K. Schmidt:** resources, data curation, supervision.

**All authors:** writing – review & editing.

## Conflict of interest statement

None declared.
