# Supplementary material for: Incorporating progesterone receptor expression into the PREDICT breast prognostic model
Source: Eur J Cancer. 2022 Sep;173:178–93. doi: 10.1016/j.ejca.2022.06.011 (PMC10412460; doi:10.1016/j.ejca.2022.06.011)
Supplement: Multimedia component 2 [file mmc2.docx]

**Supporting Information**

**Table S1.** Excel spreadsheet with details from the BCAC studies with breast cancer patients of European descent

**Table S2.** Cumulative observed vs predicted breast cancer deaths at up to 15 years follow up by ethnicity in the New Zealand cohort. Patients who underwent chemotherapy were assumed to have been treated with anthracycline-based regimen (second-generation regimen).

|  | **Total number of breast cancer patients by ethnic group** | **Predicted breast cancer specific mortality** | | **Observed breast cancer specific mortality** |
| --- | --- | --- | --- | --- |
|  |  | **Without PR status** | **With PR status** |  |
| **Number of deaths** |  |  |  |  |
| Maori | 1054 | 118 | 118 | 108 |
| Pacific | 666 | 92 | 92 | 70 |
| European | 8220 | 807 | 816 | 695 |
| Other | 1257 | 122 | 123 | 66 |
| Missing | 168 | 14 | 14 | 1 |
| Total | 11365 | 1153 | 1163 | 940 |
| **ER specific** |  |  |  |  |
| ER- |  |  |  |  |
| Maori | 177 | 53 | 53 | 44 |
| Pacific | 153 | 44 | 45 | 31 |
| European | 1576 | 368 | 371 | 281 |
| Other | 258 | 59 | 59 | 35 |
| Missing | 30 | 8 | 8 | 1 |
| Total | 2194 | 532 | 536 | 392 |
| ER+ |  |  |  |  |
| Maori | 877 | 65 | 65 | 64 |
| Pacific | 513 | 48 | 47 | 39 |
| European | 6644 | 439 | 445 | 414 |
| Other | 999 | 64 | 65 | 31 |
| Missing | 138 | 6 | 6 | 0 |
| Total | 9171 | 622 | 628 | 548 |

**Table S3.** Cumulative observed vs predicted breast cancer deaths at up to 15 years follow up by ethnicity in the New Zealand cohort. Patients with positive HER2 tumours were assumed to have been treated with trastuzumab.

|  | **Total number of breast cancer patients by ethnic group** | **Predicted breast cancer specific mortality** | | **Observed breast cancer specific mortality** |
| --- | --- | --- | --- | --- |
|  |  | **Without PR status** | **With PR status** |  |
| **Number of deaths** |  |  |  |  |
| Maori | 1054 | 111 | 111 | 108 |
| Pacific | 666 | 85 | 85 | 70 |
| European | 8220 | 767 | 776 | 695 |
| Other | 1257 | 115 | 116 | 66 |
| Missing | 168 | 13 | 13 | 1 |
| Total | 11365 | 1092 | 1101 | 940 |
| **ER specific** |  |  |  |  |
| ER- |  |  |  |  |
| Maori | 177 | 48 | 48 | 44 |
| Pacific | 153 | 39 | 40 | 31 |
| European | 1576 | 345 | 348 | 281 |
| Other | 258 | 54 | 54 | 35 |
| Missing | 30 | 7 | 7 | 1 |
| Total | 2194 | 494 | 498 | 392 |
| ER+ |  |  |  |  |
| Maori | 877 | 62 | 62 | 64 |
| Pacific | 513 | 46 | 45 | 39 |
| European | 6644 | 422 | 428 | 414 |
| Other | 999 | 62 | 62 | 31 |
| Missing | 138 | 6 | 6 | 0 |
| Total | 9171 | 598 | 603 | 548 |

**Figure S1.** Plot with the hazard ratio of PR status per study and adjusted for the prognostic index for studies with at least 2 PR-negative and 2 PR-positive breast cancer deaths

1. **ER-negative patients**


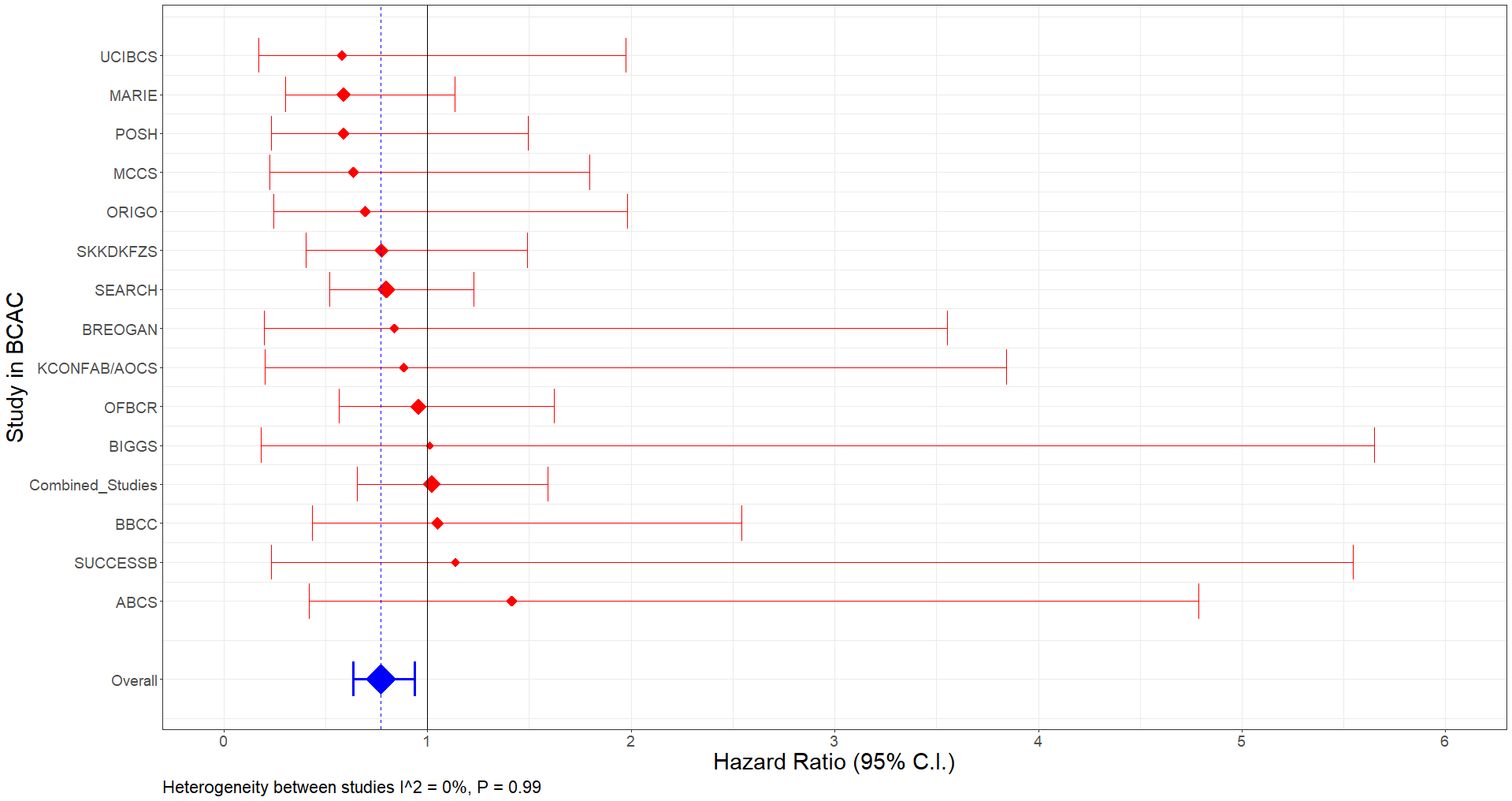


1. **ER-positive patients**


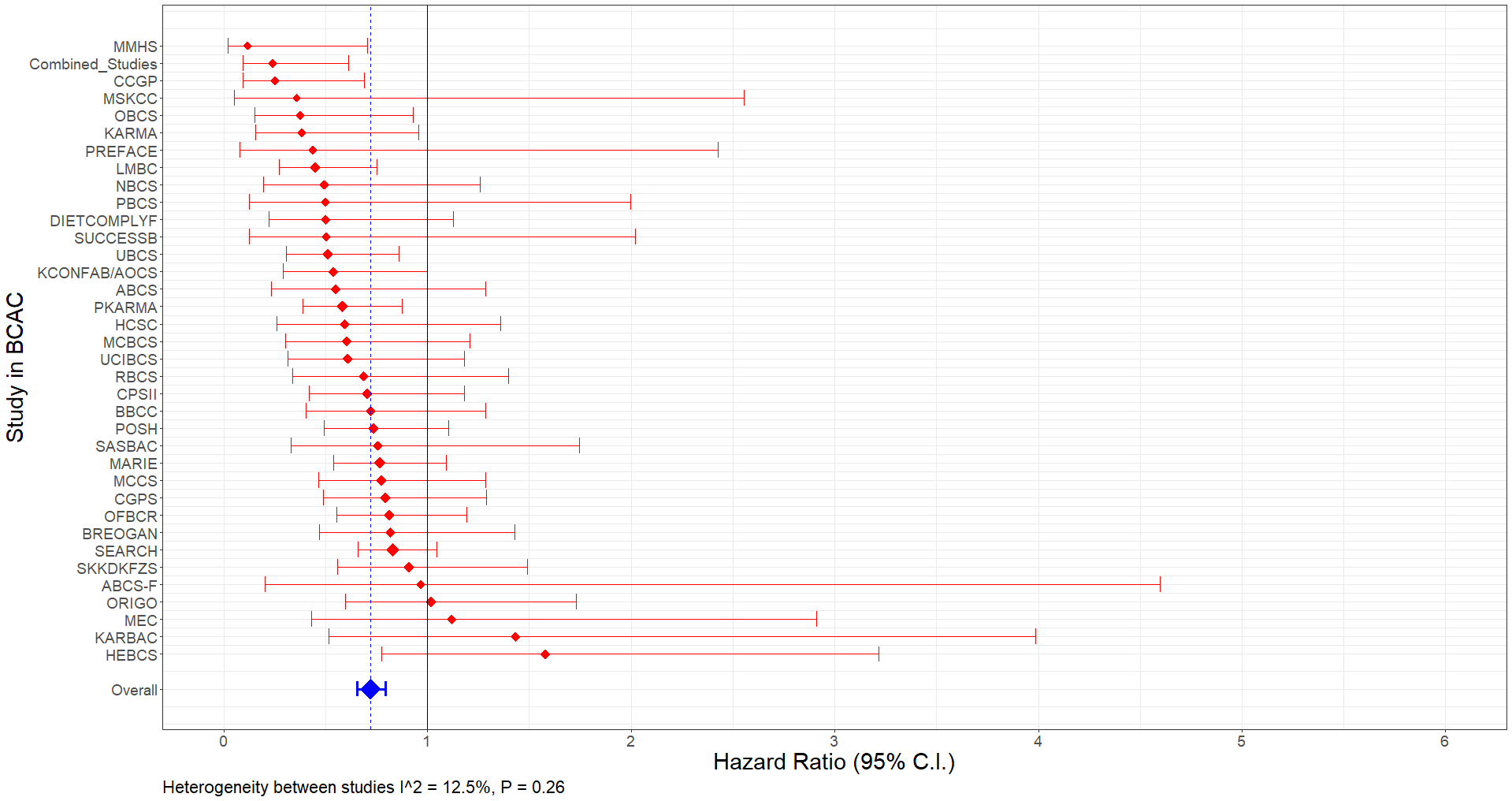


**Figure S2.** Assessment of the proportional hazard assumption using log-cumulative hazard plot for PR status stratified by ER status

**
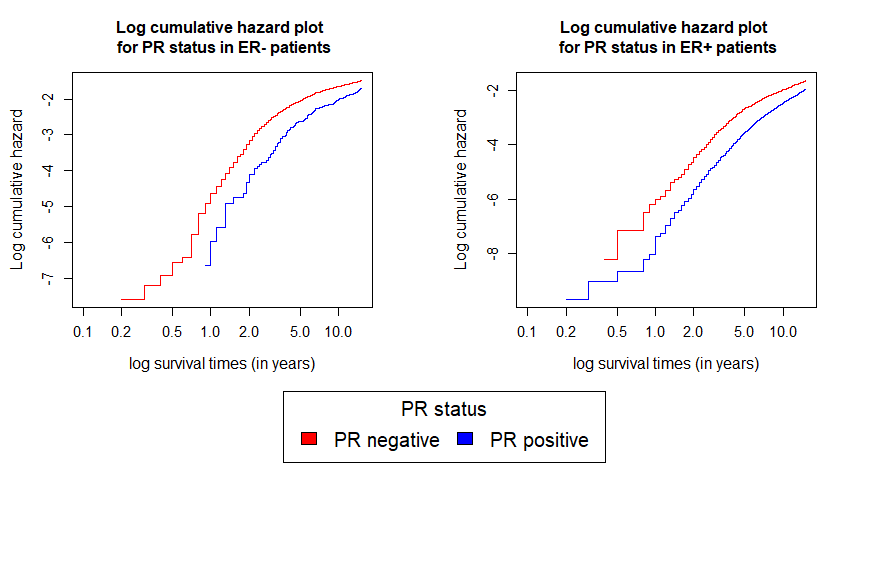
**

**Figure S3.** Assessment of the proportional hazard assumption using Schoenfeld Residuals plot for PR status stratified by ER status

1. *ER-negative patients*

*
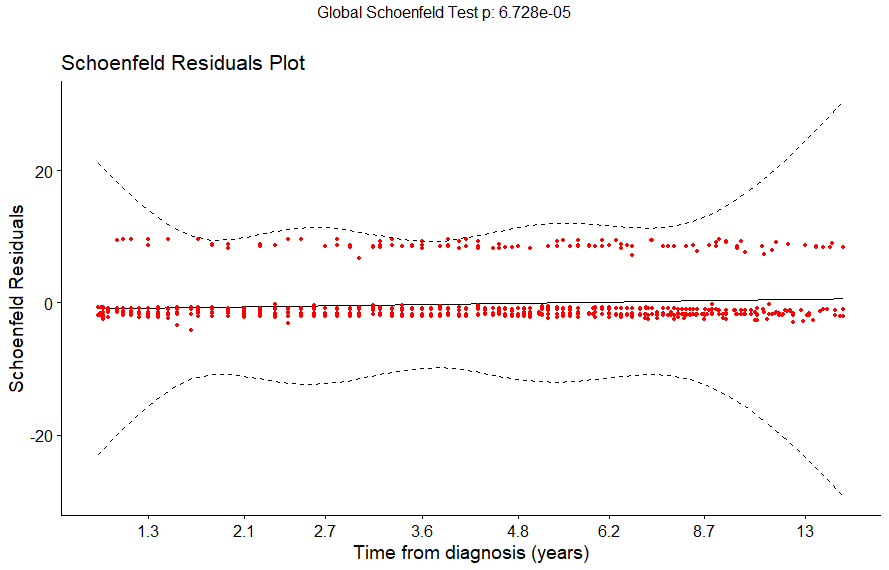
*

1. *ER-positive patients*

*
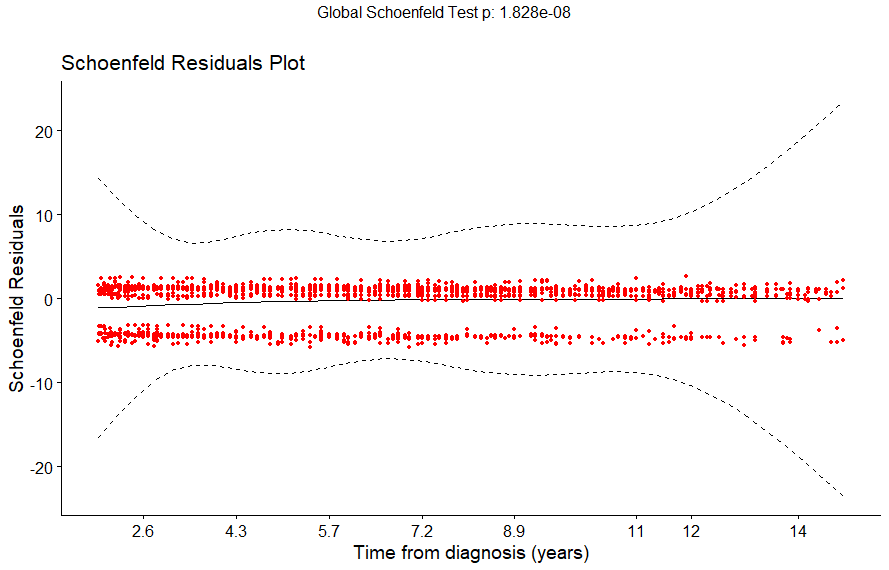
*
